# Prevalence of Pulmonary Hypertension in Liver Cirrhosis Patients: A Cross-Sectional Analytical Study

**DOI:** 10.7759/cureus.57313

**Published:** 2024-03-31

**Authors:** Augustine A Enenche, Anthony G Kweki, Henry O Aiwuyo, Oluwasegun M Akinti, Anna Nevolina, Jamal C Perry, Yonael Ayinalem, John O Osarenkhoe, Emmanuel Ukenenye, Charles O Poluyi, Aishatu O Ibrahim

**Affiliations:** 1 Internal Medicine, Jos University Teaching Hospital, Jos, NGA; 2 Internal Medicine/Cardiology, Dalhatu Araf Specialist Hospital, Lafia, NGA; 3 Internal Medicine/Cardiology, Colchester Hospital, East Suffolk and North Essex NHS Foundation Trust (ESNEFT), Colchester, GBR; 4 Internal Medicine, Brookdale University Hospital Medical Center, Brooklyn, USA; 5 Medicine, Brookdale University Hospital Medical Center, Brooklyn, USA; 6 Medicine and Surgery, Igbinedion University Teaching Hospital, Benin City, NGA; 7 Internal Medicine/Cardiology, Jos University Teaching Hospital, Jos, NGA

**Keywords:** hepatopulmonary syndrome, cross-sectional analytical study, echocardiography, liver cirrhosis, pulmonary hypertension

## Abstract

Background: Liver cirrhosis (LC) is a common complication of chronic liver disease. Its prevalence has increased markedly over the last few years. With liver cirrhosis comes cardiovascular morbidity and mortality. It is important that the detection of the abnormalities by echocardiography be given priority, as this can change the clinical outcome of these patients with cardiovascular abnormalities in liver cirrhosis.

Aim: This study aims to determine the prevalence of pulmonary hypertension in LC patients.

Methods and materials: A cross-sectional analytical study was carried out at JUTH (Jos University Teaching Hospital) over a period of one year. We recruited 210 adult patients with liver cirrhosis from the gastroenterology clinic and wards for this study. Data from these patients were collected with questionnaires administered by the interviewer and analysed using SPSS 23 statistical software (IBM Corp., Armonk, NY). The data obtained are presented in tables and charts. Categorical variables were expressed as proportions and frequencies, while continuous data were expressed as the median, mean, and standard deviation.

Results: Pulmonary hypertension was found in 30.5% of the participants, with mild pulmonary hypertension being the most common. No one had severe pulmonary hypertension. There was an increased risk of developing pulmonary hypertension in patients with coughs, easy fatigability, bilateral leg swelling, abdominal swelling, and ascites (P<0.05).

Conclusion: The result showed that there is a high prevalence of pulmonary hypertension in patients with liver cirrhosis.

## Introduction

Liver cirrhosis (LC), a complication of chronic liver disease, is defined as a progressive, diffuse fibrosing nodular condition that disrupts the entire normal liver architecture [1,2].

Liver cirrhosis is recorded as the 11th cause of death globally, and over 1 million deaths have been recorded from complications of liver disease [3]. Liver cirrhosis occurs globally. However, age groups, sex, ethnicity, and geographical locations influence its incidence and prevalence [4,5]. It is ranked as the fifth most common cause of death in the United Kingdom and Mexico [4,6]. The common causes of liver cirrhosis include alcoholic and non-alcoholic steatohepatitis, as well as viral hepatitis [4]. Other notable causes include Wilson’s disease, hemochromatosis, cardiac cirrhosis, and biliary cirrhosis [7].

Cardiovascular changes can complicate LC. Pulmonary hypertension, left and right ventricular systolic and diastolic dysfunction, coronary artery disease, and cirrhotic cardiomyopathy are among these cardiovascular changes [8-12].

The two main pulmonary complications of LC include pulmonary hypertension and hepatopulmonary syndrome [13]. Pulmonary hypertension usually occurs in the setting of portal hypertension, which is one of the complications of LC [13]. Pulmonary hypertension is defined as a pulmonary pressure greater than 25 mmHg at rest or 30 mmHg during exercise [13]. Pulmonary hypertension is observed to be due to endothelial remodelling and vasoconstriction in patients with LC, which occurs in about 5-10% of patients undergoing liver transplants [13,14]. Donovan et al. [9] discovered a 12% prevalence in Michigan among patients preparing for a liver transplant. The higher the pulmonary pressure, the higher the risk of death in LC patients undergoing liver transplantation [13]. Therefore, liver cirrhosis patients undergoing liver transplantation with pulmonary hypertension ought to be treated with vasodilators, endothelin blockers, or phosphodiesterase inhibitors to reduce the pulmonary pressure before the transplant [13]. Pulmonary hypertension is not common among those with LC from hepatitis C viral infection; it was an observational finding [14]. Echocardiography has been recommended for screening in patients billed for liver transplants to rule out pulmonary hypertension [14]. Stephanie and Tisha [11] showed that pulmonary hypertension can be treated with vasodilators, successfully reducing mortality. Mota and Filho [15] have published that cardiac catheterization is the gold standard for measuring pulmonary pressure, but transthoracic echocardiography has 100% sensitivity and 80% specificity and thus can be used to measure pulmonary pressure.

Hepatopulmonary syndrome is characterized by pulmonary vascular dilatation, physiologic shunting with resultant ventilation-perfusion mismatch, and hypoxaemia in patients with liver cirrhosis [13,16]. It occurs in 20% of LC patients awaiting liver transplantation; 5% of these patients have clinical hypoxaemia, which is due to intrapulmonary shunting [11,14,16]. Criteria for making a diagnosis of hepatopulmonary syndrome include the presence of liver cirrhosis, hypoxia, and intrapulmonary shunts [11,16]. Five-year survival is about 70% following treatment with vasodilators and liver transplants [11].

There is a progressive rise in pulmonary pressure in patients with liver cirrhosis, which increases the risk of death [11,15,17]. The prevalence of hepatopulmonary syndrome is between 17% and 47%, and its natural history is characterized by progressive clinical deterioration with severe dyspnoea and high mortality after four to five years from the first respiratory symptom [5,18]. The degree of liver disease does not predict the presence or severity of hepatopulmonary syndrome or hypoxemia [11,16].

This study provides an opportunity to determine the pulmonary abnormalities in patients with liver cirrhosis, thus reducing the dearth of knowledge about cardiovascular complications of liver cirrhosis.

## Materials and methods

The study was conducted in adults with liver cirrhosis at Jos University Teaching Hospital (JUTH). Jos is the capital of Plateau State in north-central Nigeria. The study was a hospital-based cross-sectional analytical study that was carried out within a 12-month period. Participants for this study were recruited from patients diagnosed with liver cirrhosis attending a gastroenterology clinic or admitted to the wards at JUTH.

The sample size was determined using Bennet Fisher’s formula [19]. An estimated sample size of 210 was obtained. A convenient sampling method was used to select the participants who would constitute the sample size.

Ethical approval was obtained from the Human Research and Ethics Committee of JUTH with approval number JUTH/DCS/IREC/127/XXX/2096.

Questionnaires, which were administered by the interviewer, were used to collect data from patients as regards the following: sociodemography, history suggestive of liver cirrhosis, and symptoms of cardiac dysfunction. A detailed examination was also conducted on each patient.

Systolic pulmonary artery pressures (SPAP) were estimated using the peak tricuspid regurgitant (TR) velocity and the simplified Bernoulli equation, combining this value with the estimate of the right atrial (RA) pressure.

$Right Ventricular Systolic Pressure = 4 * TR Velocity (V^{2}) + RA Pressure$

Right atrial pressure was estimated from the inferior vena cava (IVC) diameter at peak expiration and the presence of inspiratory collapse. IVC ≤ 2.1 cm that collapses >50% with a sniff suggests a normal RA pressure of 3 mmHg (range, 0-5 mmHg), whereas an IVC diameter >2.1 cm that collapses <50% with a sniff suggests a high RA pressure of 15 mmHg (range, 10-20 mmHg). In indeterminate cases in which the IVC diameter and collapse do not fit this paradigm, an intermediate value of 8 mmHg (range, 5-10 mmHg) was used [20]. Mild pulmonary hypertension is defined as a mean pulmonary arterial pressure (mPAP) of 25-34.9 mmHg, moderate as a mPAP of 35-44.9 mmHg, and severe as a mPAP >45 mmHg [21]. The mean pulmonary artery pressure was estimated using the Chemla formula mPAP = (0.61 × SPAP) + 2 [21].

The data obtained were analysed with the SPSS 23 statistical software. The data obtained are presented in tables and charts. Categorical variables were expressed as proportions and frequencies, while continuous data were expressed as median, mean, and standard deviation.

## Results

Social demographic characteristics

As shown in Table 1, the mean age of the participants was 47 ± 12.47 years. More than half of the participants were males, 142 (67.6%), and 68 (32.4%) were females. Only 78 (37.1%) had secondary education; 60 (28.6%) had tertiary education; 40 (19.0%) had primary education; 4 (1.9%) had informal education; and 28 (13.3%) had no form of education. The most common occupations among the participants were farming and civil service, with 60 (28.6%) each, and trading was next, accounting for 42 (20.0%) of the study population. Most of the participants reside in urban settlements, 118 (56.2%), while 92 (44.8%) reside in rural areas.

**Table 1:** Demographic characteristics of patients with liver cirrhosis

Demographic characteristics	Frequency	Percentage %
Mean ± SD age years	47±12.47	
Gender		
Male	142	67.6
Female	68	32.4
Educational level		
None	28	13.3
Informal	4	1.9
Primary	40	19.0
Secondary	78	37.1
Tertiary	60	28.6
Occupation		
Student	4	1.9
Civil servant	60	28.6
Trader	42	20.0
Farmer	60	28.6
House-wife	8	3.8
Retiree	8	3.8
Artisan	12	5.7
Others	16	7.6
Residence		
Urban	118	56.2
Rural	92	43.8

Clinical characteristics

Table 2 shows that abdominal swelling was the most common clinical symptom, present in 120 (57.1%) of the participants. Easy fatigability was reported in 96 (45.7%), while 84 (40.0%) had ascites. Pedal swelling was seen in 80 (38.1%). Only 4 (1.9%) of the participants had orthopnoea and paroxysmal nocturnal dyspnoea, respectively.

**Table 2:** Clinical characteristics of patients with liver cirrhosis Note: multiple abnormal heart sounds were heard in a single patient

Variable	Yes (%)	No (%)
Orthopnoea	4(1.9)	206(98.1)
Paroxysmal nocturnal dyspnoea	4(1.9)	206(98.1)
Cough	24(11.4)	186(88.6)
Chest pain	20(9.5)	190(90.5)
Palpitation	12(5.7)	198(94.3)
Easy fatigability	96(45.7)	114(54.3)
Bilateral leg swelling	80(38.1)	130(61.9)
Abdominal swelling	120(57.1)	90(42.9)
Ascites	84(40.0)	126(60.0)
Intermittent claudication	6(2.9)	204(97.1)

Prevalence of pulmonary hypertension

Pulmonary hypertension was found in 64 (30.5%) of the participants, as shown in Table 3. Of the 64 (30.5%) with pulmonary hypertension, 54 (25.75%) had mild pulmonary hypertension, while 10 (4.8%) had moderate pulmonary hypertension. None of the participants had severe pulmonary hypertension (Table 3).

**Table 3:** Prevalence and grade of pulmonary hypertension in patients with liver cirrhosis

	Frequency (N)	Percentage (%)
Pulmonary hypertension		
Yes	64	30.5
No	146	69.5
Grades of pulmonary hypertension		
None	146	69.5
Mild	54	25.7
Moderate	10	4.8

In Table 4, logistic regression of the relationship between clinical symptoms and the presence of pulmonary hypertension showed an increased risk for pulmonary hypertension with complaints of cough, easy fatigability, bilateral leg swelling, abdominal swelling, and ascites (P-values<0.05).

**Table 4:** Logistic regression showing the relationship between clinical symptoms and the presence of pulmonary hypertension OR: odd ratio, CI: confidence interval *P-values < 0.05 is significant

	Unstandardized coefficients		OR	95% CI		p-value
	B	S.E.		Lower	Upper	
Orthopnoea	0.843	1.011	2.323	0.320	16.863	0.405
Paroxysmal nocturnal dyspnoea	0.843	1.011	2.323	0.320	16.863	0.405
Cough	1.337	0.446	3.808	1.589	9.124	0.003*
Chest pain	−0.613	0.580	0.542	0.174	1.690	0.291
Palpitation	0.140	0.632	1.150	0.333	3.966	0.825
Easy fatigability	0.796	0.306	2.218	1.218	4.036	0.009*
Bilateral leg swelling	0.527	0.305	1.694	0.931	3.081	0.084*
Abdominal swelling	0.706	0.316	2.026	1.090	3.768	0.026*
Ascites	0.777	0.305	2.176	1.196	3.958	0.011*
Intermittent claudication	0.136	0.879	1.145	0.204	6.417	0.877

## Discussion

Pulmonary hypertension was found in 30.5% of the patients recruited. Most of the participants with pulmonary hypertension had mild pulmonary hypertension, and only 4.8% of them had moderate pulmonary hypertension. None of the patients had severe pulmonary hypertension. Various studies have been carried out to assess the pulmonary pressure of patients with liver cirrhosis. Gurghean and Tudor [22] found pulmonary hypertension in 27% of patients with hepatic cirrhosis. An online source reported a prevalence of 20% in patients with liver disease [23]. Donovan et al. [9] found a prevalence of 12%. Studies have shown that pulmonary hypertension has an adverse effect on the outcome of patients with liver cirrhosis; the higher the pulmonary pressures, the greater the risk of death in individuals undergoing liver transplants [13]. The difference observed in various studies could be attributed to geographical location. All the above studies were done outside the West African sub-region.

Echocardiography currently plays an important role in the diagnosis of pulmonary hypertension [24]. It has the added benefit of being non-invasive and readily available [24]. Echocardiographic parameters are closely related to pulmonary hemodynamics. It allows assessment of the cardiac structure and function and estimation of the pressure in the right ventricle, right atrium, and pulmonary trunk; it also excludes other causes of elevated pulmonary bed pressure [24]. Echocardiography has been reported to have a good correlation and agreement with right heart catheterization in estimating systolic pulmonary artery pressure; therefore, it is appropriate for screening patients because of its high sensitivity [25]. The index study, as well as that of Gurghean and Tudor [22], employed echocardiography in the diagnosis of pulmonary hypertension.

The index study observed an increased risk of pulmonary hypertension in patients with liver cirrhosis with complaints of cough, easy fatigability, bilateral leg swelling, abdominal swelling, and ascites. Hoeper et al. [26] reported similar clinical findings as cardinal symptoms of pulmonary hypertension; this is manifested as progressive exercise dyspnea, often accompanied by fatigue and exhaustion. In the phase of cardiac decompensation, which is also seen in cirrhotic liver disease, there is a rise in the right cardiac filling pressures; as such, the typical triad of cervical venous congestion, ascites, and oedema occurs [26,27].

However, clinical features are unspecific, which can result in a delay of many months or even years between the onset of symptoms and the diagnosis of pulmonary hypertension [26]. However, a high index of suspicion or routine echocardiography for assessing pulmonary hypertension in patients with cirrhotic liver disease will be beneficial for initiating early treatment and, therefore, improving the management outcome.

The study is limited to the following: first, only two-dimensional echocardiography was used to carry out this study; three-dimensional echocardiography would have provided an opportunity to do a volumetric study. More so, right heart catheterization, the gold standard for the diagnosis of pulmonary hypertension, was not done for any of the patients. Additionally, the study was unable to prognosticate patients with liver cirrhosis based on the cardiovascular parameters obtained.

## Conclusions

Pulmonary hypertension is a common finding among these patients; the degree of pulmonary hypertension found in these patients is mild and moderate. None of the patients were found to have severe pulmonary hypertension. Echocardiography is, therefore, an important screening modality for detecting cardiovascular morbidities such as pulmonary hypertension in liver cirrhotic patients.

## Appendices

Questionnaire

The questionnaire administered by the interviewer, which was used to collect data from patients, is shown in Table 5.

**Table 5:** Questionnaire: Echocardiographic assessment of right ventricular function in adults with liver cirrhosis at Jos University Teaching Hospital

Date															
Serial number															
Socio-demographic data															
I.	Initials														
II.	Hospital number														
III.	Sex (tick)			Male ( )				Female ( )							
IV.	Occupation			Student ( )				Civil servant ( )							Trader ( )
	Farmer ( )			Others - specify...................											
V.	Marital status				Single ( )			Divorced ( )							Married ( )
	Widowed( )														
VI	Educational level						None ( )			Informal ( )		Primary ( )			
	Secondary ( )						Tertiary ( )								
VIII		Residence				Urban ( )					Rural ( )				
Clinical history															
I.	Orthopnoea		yes ( )						no ( )					If yes duration........	
II.	Paroxysmal nocturnal dyspnoea		yes ( )						no ( )					If yes duration........	
III.	Cough		yes ( )						no ( )				If yes duration........		
IV.	Chest pain		yes ( )						no ( )				If yes duration........		
V.	Palpitation		yes ( )						no ( )					If yes duration........	
VI.	Easy fatigability		yes ( )						no ( )					If yes duration........	
VII.	Bilateral leg swelling		yes ( )						no ( )					If yes duration........	
VIII	Abdominal swelling		yes ( )						no ( )					If yes duration........	
IX	Intermittent claudication		yes ( )						no ( )					If yes duration........	
